# Predictive uncertainty in auditory sequence processing

**DOI:** 10.3389/fpsyg.2014.01052

**Published:** 2014-09-23

**Authors:** Niels Chr. Hansen, Marcus T. Pearce

**Affiliations:** ^1^Music in the Brain, Center of Functionally Integrative Neuroscience, Department of Clinical Medicine, Aarhus University HospitalAarhus, Denmark; ^2^Royal Academy of Music Aarhus/AalborgAarhus, Denmark; ^3^Department of Aesthetics and Communication, Aarhus UniversityAarhus, Denmark; ^4^Cognitive Science Research Group, School of Electronic Engineering and Computer Science, Queen Mary University of LondonLondon, UK

**Keywords:** statistical learning, information theory, entropy, expectation, auditory cognition, music, melody

## Abstract

Previous studies of auditory expectation have focused on the expectedness perceived by listeners retrospectively in response to events. In contrast, this research examines *predictive uncertainty*—a property of listeners' prospective state of expectation prior to the onset of an event. We examine the information-theoretic concept of Shannon entropy as a model of predictive uncertainty in music cognition. This is motivated by the Statistical Learning Hypothesis, which proposes that schematic expectations reflect probabilistic relationships between sensory events learned implicitly through exposure. Using probability estimates from an unsupervised, variable-order Markov model, 12 melodic contexts high in entropy and 12 melodic contexts low in entropy were selected from two musical repertoires differing in structural complexity (simple and complex). Musicians and non-musicians listened to the stimuli and provided explicit judgments of perceived uncertainty (explicit uncertainty). We also examined an indirect measure of uncertainty computed as the entropy of expectedness distributions obtained using a classical probe-tone paradigm where listeners rated the perceived expectedness of the final note in a melodic sequence (inferred uncertainty). Finally, we simulate listeners' perception of expectedness and uncertainty using computational models of auditory expectation. A detailed model comparison indicates which model parameters maximize fit to the data and how they compare to existing models in the literature. The results show that listeners experience greater uncertainty in high-entropy musical contexts than low-entropy contexts. This effect is particularly apparent for inferred uncertainty and is stronger in musicians than non-musicians. Consistent with the Statistical Learning Hypothesis, the results suggest that increased domain-relevant training is associated with an increasingly accurate cognitive model of probabilistic structure in music.

## Introduction

Recent theories of cognition propose that the human brain is adapted for making correct predictions about the future (Bar, 2007, 2011) in order to enhance survival by regulating arousal and directing attention (Bubic et al., 2010). This idea has its roots in von Helmholtz' (1870/1985) argument that predictions based on prior experience affect how we perceive the world around us. Since then, empirical and theoretical research has provided support for this view across a wide variety of domains including language comprehension (DeLong et al., 2005), decision making (Platt and Glimcher, 1999), motor processing (Wolpert and Flanagan, 2001), and visual perception (Egner et al., 2010). Theories of brain function in perceptual processing propose that increasingly accurate predictive models of the environment result from continuous optimization of cognitive and neural representations of the sensorium resulting from violation and confirmation of expectations (Friston, 2005, 2009, 2010). The notion that learning emerges from changes in synaptic weights gains further support from accumulating evidence that different types of neuronal plasticity (Stiles, 2001)–and even neurogenesis (Gross, 2000; Taupin, 2006)–persist throughout adulthood.

The present study elaborates on previous research by examining the cognitive mechanisms underlying *predictive uncertainty*, defined as characterizing the strength and specificity of an individual's expectations over the range of *possible* events, generated *before* the next event actually happens. Expectation is a process of generating predictions about future events. Predictive uncertainty describes a cognitive state before the event in question occurs, and varies as a function of how subjectively likely, or expected, the different possible outcomes are. So expectedness refers to the likelihood of individual events, which may or may not have happened yet, while uncertainty is a property of a collection of expectedness judgements about the different possible outcomes for a future event (e.g., what is the pitch of the next note). For example, a listener who is sure that the pitch of the next note in a melody will be a middle C (261.63 Hz) and not any other note has low predictive uncertainty at that point in time (before the next note is heard); another listener to the same melody who expects to hear any pitch between middle C and the A above it (440 Hz) with equal likelihood, has high predictive uncertainty. Note that predictive uncertainty is distinct from the expectedness of the event that actually happens. Our first listener is highly certain about the next event in the melody but may be incorrect (and surprised) if the note that actually follows is not a middle C.

Empirical research suggests that individuals readily learn the probabilistic structure of sequential sensory input through implicit statistical learning during exposure and are able to generalize this learning to new examples. In psycholinguistics this has been formulated as a *Statistical Learning Hypothesis*, according to which word segmentation follows frequency distribution in received language input (Cristià et al., 2011). In addition to studies of language acquisition (Saffran et al., 1996; see review by Saffran, 2003a), statistical learning has been demonstrated for sequences of abstract visual shapes (Fiser and Aslin, 2002; Kirkham et al., 2002), animal pictures (Saffran et al., 2007), synthesized instrumental timbres (Tillmann and McAdams, 2004), sine tones in familiar (Saffran et al., 1999) and unfamiliar musical systems (Loui et al., 2010), interleaved melodies (Creel et al., 2004), serialist music (Dienes and Longuet-Higgins, 2004) as well as for visuomotor sequences (Hunt and Aslin, 2001) and sequential patterns of tactile finger stimulation (Conway and Christiansen, 2005). The diversity of the stimuli in these studies has led researchers to argue that statistical learning represents a domain-general mechanism (Kirkham et al., 2002; Perruchet and Pacton, 2006), albeit with domain-specific biases (Saffran, 2003b), possibly partly due to modality-specific neural implementations of the same mechanism (Conway and Christiansen, 2005, 2006). Here, we investigate whether the output of such implicit statistical learning might account for individuals' predictive uncertainty when processing sensory input.

We focus on auditory processing, and music in particular, as a convenient stimulus for examining this question. First, music is a complex, multidimensional, sequential auditory stimulus that exists in all human cultures. Effects of long-term learning can be examined with musical stimuli because nearly everyone has extensive exposure to music throughout their lives (even if only incidentally). Second, we have large digital corpora of music enabling automatic, data-rich analysis of probabilistic features (e.g., the Essen Folk Song Collection, see Section Stimuli). We know of no comparable databases for modeling of long-term exposure to visual sequences. Large datasets of spoken language do exist; however, music has the advantage that it lacks referential semantics, thus removing a layer of complexity from the analysis of statistical learning. For these reasons, we focus on predictive uncertainty in music perception in the following sections.

### Statistical learning of musical structure

Empirical support for the Statistical Learning Hypothesis within the musical domain has been reported using various behavioral procedures. In a short-term statistical learning experiment, Saffran et al. (1999) found that infants and adults are able to identify 3-note groups (tone words) distinguished only by transition probabilities acquired through implicit learning during 21 min of exposure to continuous sequences of concatenated tone words. Tone words are distinguished only in that transition probabilities in the exposure stream are greater within than between words. In addition to absolute pitch cues, further research using this paradigm has demonstrated sensitivity to the learned statistical properties of relative cues such as pitch interval in infant (Saffran et al., 2005) and adult listeners (Saffran and Griepentrog, 2001; Saffran, 2003c).

Other research has demonstrated that statistical regularities in progressions of musical harmonies can similarly be acquired through short-term exposure and that the degree of exposure increases listeners' ability to identify grammatical errors (Jonaitis and Saffran, 2009). Rohrmeier and Cross (2009) confirmed that implicit as well as explicit structural knowledge becomes available to the listener after short-term exposure and, furthermore, found that increasing grammatical complexity deteriorates learnability. This is consistent with an interpretation of Saffran (2003c) and Saffran and Griepentrog's (2001) findings that the complexity of the cognitive representation required for a given task (i.e., an alphabet size of 7 vs. 12) influences the learnability of probabilistic information.

In addition to findings relating to short-term encoding of regularities in the immediate context (see also Oram and Cuddy, 1995), there is evidence that pitch expectations are informed by long-term exposure. The evidence here comes mainly from probe tone studies in which listeners hear musical contexts with different final *probe tones* and are asked to rate how well the probe tone fits the context (e.g., Cuddy and Lunny, 1995; Krumhansl, 1995a,b; Schellenberg, 1996; Krumhansl et al., 1999, 2000). However, because the goal of these studies was to examine theories of pitch continuation (e.g., Narmour's, 1990, 1992 Implication-Realization Model and Brown et al., 1994 Intervallic-Rivalry Theory), their authors did not systematically manipulate the predictive uncertainty of their melodic contexts, which means that the results cannot be used to test theories of predictive uncertainty. Furthermore, because the contexts were usually selected to generate strong expectations for a single continuation (e.g., Schellenberg, 1996), the results may not generalize to expectations in highly uncertain contexts.

Providing further support for implicit statistical learning of musical structure, Krumhansl (1990) showed that tonal expectations derived from probe-tone experiments (Krumhansl and Kessler, 1982) are closely related to zeroth-order distributions of chromatic scale degrees in large collections of music. In addition, Pearce and Wiggins (2006) and Pearce et al. (2010a) demonstrated that pitch expectations generated while listening to melodies correspond closely with note probabilities estimated from a large disjoint corpus of music. Using harmonies, Tillmann and colleagues have shown that target chords are processed more accurately and quickly when they are related both to the local and the global harmonic context (i.e., previous chord and prior context of six chords, respectively) (Tillmann et al., 1998) and that these effects can be explained by a mechanism of long-term statistical learning of sequential harmonic patterns in music (Tillmann et al., 2000).

In summary, research suggests that probabilistic relationships between musical events are internalized on a short-term as well as on a long-term basis. These studies have focused on the expectedness of events perceived by listeners retrospectively after a given event has occurred. By comparison, predictive uncertainty of the cognitive process of generating expectations before the forthcoming event arrives has received little attention in research to date. An exception is Schmuckler (1989) who analyzed the uncertainty of expectedness distributions using a coarse non-probabilistic measure of the difference between average and maximum expectedness ratings. Thus, while it has been established that the expectedness of musical events reflects internalized subjective probabilities, it has yet to be ascertained which properties of probability distributions give rise to different states of predictive uncertainty.

### Information theory and uncertainty in music cognition

Information theory (Shannon, 1948) provides powerful tools for quantifying expectedness and uncertainty in terms of the information content and entropy of probability distributions, respectively. Given a random variable *X* with a discrete set of possible events occurring with probabilities *p*(*x*_1_), *p*(*x*_2_), …, *p*(*x_n_*), Shannon defined the information content of an event *x_i_* as:

$$IC\left(x_{i}\right)=\;-log_{2}\;p(x_{i})$$

Information content is thus inversely proportional to probability (MacKay, 2003, p. 32) and reflects the unexpectedness of the event.

Entropy (*H*), on the other hand, measures the uncertainty involved in predicting the outcome of *X* as the expected value of the information content:

$$H\left(X\right)=\;-\sum_{i\;=\;1}^{n}p\left(x_{i}\right)\;log_{2}\;p(x_{i})\;$$

*H* is measured in bits, and it is assumed that probabilities sum to unity, ∑ *p*(*x_i_*) = 1, and that no probabilities equal zero, *p*(*x_i_*) > 0. Maximum entropy results when all possible events are equiprobable, *p*(*x_i_*) = 1/*n* where *H_max_* = log_2_ *n*, with *n* representing the alphabet size of *X*. The normalized entropy, *H_norm_* = *H/H_max_*, is sometimes preferred, because it is comparable across distributions varying in alphabet size.

Empirical psychological research has used Shannon entropy to understand cognitive processes involved in, for example, sentence comprehension (Hale, 2006), anxiety (Hirsh et al., 2012), consciousness (Carhart-Harris et al., 2014), and strategy choice in decision making (Swait and Adamowicz, 2001). In the musical domain, Meyer (1957) pioneered the application of an information-theoretic framework to theories of music cognition. His statement that “musical styles are internalized probability systems” (p. 414) spurred a comprehensive research program using entropy as a delimiter of musical style (Youngblood, 1958; Siromoney and Rajagopalan, 1964; Hiller and Bean, 1966; Hiller and Ramon, 1967; Zanten, 1983; Margulis and Beatty, 2008) and, more recently, for addressing issues in music information retrieval (Madsen and Widmer, 2007a,b; Duane, 2010). Importantly, none of these studies have examined entropy as a cognitive model of predictive uncertainty, focusing instead on engineering applications (e.g., melody identification) or musicological analysis (e.g., comparing the average entropy of different musical styles). As a result, many of these studies have estimated entropy from small collections of music, rather than attempting to build a cognitive model that incorporates the schematic effects of long-term exposure on expectations and predictive uncertainty.

The approach we take has two potential precursors within the music cognition literature. Although they applied it to a very different aspect of music cognition to that studied here, Desain and Honing (2003) used Shannon entropy to characterize within- and between-participant response consistency in a categorical rhythm discrimination task. In his book, *Sweet Anticipation* (2006), David Huron suggested that entropy can be used to measure the strength of melodic expectations (pp. 53–55, 154, 162). Huron summarizes an unpublished study in which he and his colleagues related the entropy of participants' bets about melodic continuations in a gamelan melody to cultural differences in expertise between American and Balinese musicians. Neither of these studies used a probabilistic model to systematically select stimuli differing in entropy (rather they used entropy only to characterize the uncertainty of listeners' responses).

### A computational model of auditory expectation

In this research, we use an information-theoretic model of auditory expectation (Pearce, 2005) to estimate the conditional probability of each note in a melody, given the preceding melodic context; we then estimate the Shannon entropy of the distribution and the information content of the note, as described above. The model learns through experience about the statistical structure of sequences and, based on this learning, its output reflects its expectations about the next event in a sequence of events to which it is exposed. Specifically, it generates a conditional probability distribution governing some attribute of the next event in a sequence of auditory events (e.g., its pitch) based on the frequency with which different pitched events have followed the current context in the past. The model output reflects both long-term schematic effects of exposure and short-term, local statistical learning. The model is based on Markov or *n*-gram methods (Manning and Schütze, 1999). The model is described and evaluated in detail elsewhere (Pearce, 2005; Pearce et al., 2010a; Omigie et al., 2012, 2013; Pearce and Wiggins, 2012) and is available for download^1^. Here we summarize some of the central features of the model, in particular three extensions to basic Markov modeling (see Pearce, 2005, for further details).

First, the model is able to vary the amount of context taken into consideration when generating the probability distributions. Basic *n*-gram models use fixed-length contexts to generate conditional probabilities of an event given the preceding events in the sequence (i.e., the probability of an event conditional on the identity of the previous *n*-1 events). Rather than using contexts of fixed-length, the present model is a variable-order Markov model (Manning and Schütze, 1999) which selects a maximum context size *k* to use, which may vary depending on the position in the melody and on its training. In making a prediction, the model combines the output of all fixed-order *n*-gram models for *n* < *k* using a weighted average in which higher-order *n*-grams are favored, a process called *smoothing* (Bunton, 1997, Manning and Schütze, 1999; Pearce, 2005, ch. 4). The maximum context size, *k*, can also be set to a particular fixed value. Using a variable-order strategy, where the maximum order used in the smoothing can vary depending on the context, improves prediction performance over fixed-order models (Pearce, 2005).

Second, the model has two components which can be used in isolation or in combination. The first component, the *long-term sub-model*, is designed to capture the effects on expectation of learning through long-term listening to music. The second, the *short-term sub-model*, is designed to capture the effects of local learning of repeated structure within a given stimulus (e.g., repeated motifs within a piece of music). The long-term sub-model is trained on a large corpus of music before being exposed to new musical pieces, while the short-term sub-model is initially empty when it is exposed to a new piece and it learns incrementally throughout listening to that piece. Here we train the long-term sub-model on a collection of folk songs and hymns which are relatively simple and strongly tonal, to simulate at a general level the musical experience of an average Western listener. The long-term and short-term sub-models each generate a conditional probability distribution for each note in the music to which they are exposed. If they are to be used in combination, these distributions are combined (using a weighted geometric mean, Pearce, 2005), yielding a single conditional distribution. Typically, the combined model shows better prediction performance than the short-term or long-term sub-models used in isolation.

Third, the model may be applied to different features of a musical sequence (e.g., pitch, onset, duration, loudness etc.) and in predicting a given feature, may combine predictions of various derived features (e.g., pitch interval, pitch contour, inter-onset interval). To achieve this, the model uses a *multiple viewpoint system* (Conklin and Witten, 1995; Pearce, 2005). We do not make extensive use of this feature in the present research, where the model predicts the pitch of the next note, using a representation in which each note in a melody consists of a pair of values: pitch interval (the difference in semitones between consecutive notes) and scale degree (the interval in semitones from the tonal center). Previous empirical research has demonstrated that the model predicts listeners' pitch expectations in a range of musical contexts, from single-interval contexts, through isochronous hymns to folk songs and chorale melodies (Pearce, 2005; Pearce et al., 2010a,b; Omigie et al., 2012).

### Evaluating entropy as a model of predictive uncertainty

The current study explores probabilistic processing as an account of the cognitive processes involved in generating expectations in musicians and non-musicians listening to two musical repertoires differing in structural complexity. Specifically, the aim is to test Shannon entropy as a model of predictive uncertainty in melodic pitch expectation. Here we use the information-theoretic model of auditory expectation described above (Pearce, 2005; see Section A Computational Model of Auditory Expectation) to select melodic contexts with high and low entropy.

Since there is little research on methods for assessing predictive uncertainty, two distinct paradigms are used here each yielding a distinct dependent variable representative of predictive uncertainty. First, *explicit uncertainty* is assessed through self-report of perceived uncertainty about what will happen next in a melody. Second, since knowledge of musical structure may not be available for explicit verbalization (Tillmann, 2005), *inferred uncertainty* is computed (using normalized entropy) from the distribution of expectedness ratings for actual continuations to each melodic context obtained using the traditional probe-tone paradigm.

Four distinct hypotheses are tested. First, following the Statistical Learning Hypothesis, it is predicted that Shannon entropy computed from probability distributions estimated through unsupervised statistical learning represents a reliable cognitive model of predictive uncertainty. Since there is no previous literature on which to base a hypothesis about possible differences between indirect and direct measures of uncertainty, main effects of entropy are expected for both inferred and explicit uncertainty.

Second, we hypothesize that individuals with high levels of domain-specific expertise (i.e., musicians) will show less predictive uncertainty on average than those with low levels of expertise (i.e., non-musicians). This reflects our proposal that training optimizes a default high-entropy cognitive model, substantiated by findings of flatter expectedness distributions (also referred to as key profiles) for non-musicians in comparison with musicians using the traditional probe-tone paradigm where probe tones follow a simple key-defining context comprising an ascending or descending major scale (Krumhansl and Shepard, 1979). Our hypothesis generalizes this effect to pitch expectations in real melodies (rather than tonal expectations in simple artificial contexts) and quantifies flatness in terms of Shannon entropy.

Third, motivated by the same proposal, we hypothesize that musicians are better able to take advantage of low-entropy contexts than non-musicians, correctly identifying low-probability continuations as such. Therefore, we predict an entropy-by-expertise interaction for the unexpectedness ratings such that musicians will show greater unexpectedness than non-musicians in low-entropy contexts (with no difference emerging in high-entropy contexts).

Fourth, as a side effect of computing inferred uncertainty from the distributions of unexpectedness ratings for individual continuations, we can replicate an established relationship between information content and perceived unexpectedness when listening to melodies (Pearce et al., 2010a) and, furthermore, test whether it generalizes across degrees of complexity and entropy. Additionally, we hypothesize from the Statistical Learning Hypothesis that this relationship will strengthen with increasing levels of expertise.

To test these hypotheses, we assess listeners' perception of predictive uncertainty when listening to musical contexts which elicit high- and low-entropy predictions. For comparison with previous research (Pearce et al., 2010a; Omigie et al., 2012), we draw half of our stimuli from a collection of isochronous hymn melodies (simple stimuli). To examine whether the findings generalize to more complex musical styles, we draw the other half of our stimuli from the vocal lines of Schubert lieder (complex stimuli). The model is used to select 24 stimuli which vary in how specifically they imply a continuation according to the model: 12 have high-entropy endings (i.e., each possible continuation tends to be equiprobable) and the other 12 have low-entropy continuations (i.e., one continuation is much more likely than the others), according to the model. We assess listeners' uncertainty in two ways. First, we simply ask them to rate on a Likert scale how uncertain they are about what will happen next in the music (*explicit uncertainty*). Second, we ask them to rate on a Likert scale how expected they find actual single-note continuations to each melody; to the extent that the distribution of expectedness ratings for the continuation tones is flat, we can infer how uncertain the listeners were about the continuation (*inferred uncertainty*). We compare the responses of a group of musicians with a group of non-musicians.

## Materials and methods

### Participants

Seventeen musicians (9 females; mean age: 26.65 years, *SD*: 5.68, range: 19–39) and 17 non-musicians (8 females; mean age: 28.94 years, *SD*: 6.42, range: 21–48) were recruited for the experiment. Members of the musician group self-declared as such and scored ≥33 on the musical training subscale of the Goldsmiths Musical Sophistication Index (Gold-MSI, v0.9) (Müllensiefen et al., 2011), whereas members of the non-musician group self-declared as such and scored ≤ 20. These upper and lower limits correspond to the 67th and 33rd percentile scores from a random sample of 488 individuals from the general British population (Müllensiefen et al., 2011). Average musical training scores for musicians and non-musicians were 53.12 (*SD*: 7.83; range: 36–62) and 13.94 (*SD*: 3.56; range: 9–20), respectively. An unpaired *t*-test with Welch's correction established a highly significant group difference [*t*_(22.36)_ = −18.79; *p* < 0.001]. The groups did not differ significantly in age [*t*_(32)_ = 1.11; *p* = 0.274] or gender [χ^2^_(1)_ = 0.12; *p* = 0.732].

### Stimuli

**Figure 1:**
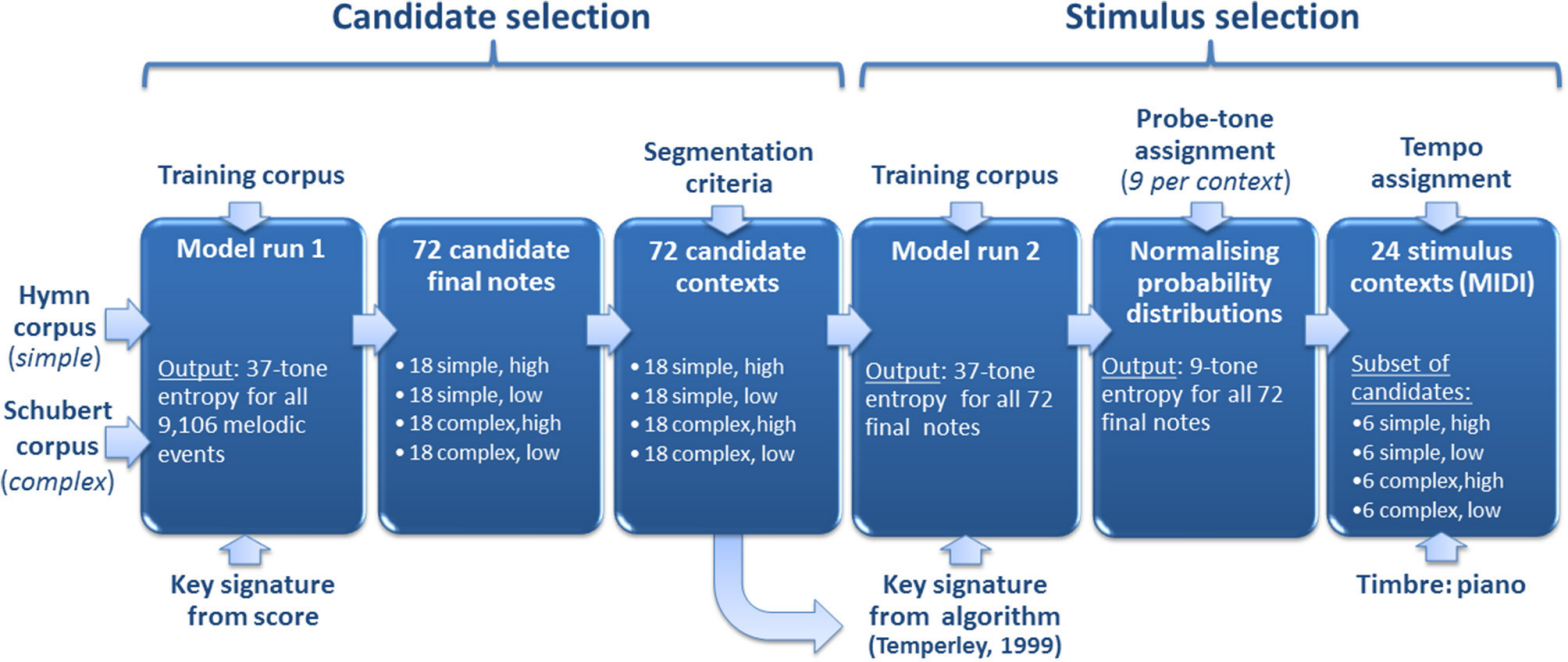
**Stimulus selection**. First, two corpora of stylistically *simple* (i.e., isochronous English hymns) and *complex* (i.e., lieder by Franz Schubert) music were analyzed by our computational model, resulting in entropy estimates for all 9106 melodic events. Second, the 18 highest and lowest scoring notes in each corpus gave rise to 72 (18 × 4) candidate melodic contexts by way of a segmentation procedure. Third, the computational model was run on the candidate contexts, resulting in updated entropy estimates. Fourth, a subset of 24 stimulus contexts was selected for use in the four conditions of the experiment (i.e., “simple, high entropy,” “simple, low entropy,” “complex, high entropy,” and “complex, low entropy”). This final selection procedure used entropy estimates based on normalized probability distributions over nine chromatically distributed continuation probe tones for each context.

The procedure of selecting stimuli for the listening experiment is outlined in Figure 1 and will be described in detail below. Stimuli were based on two musical corpora differing in rhythmic and tonal complexity: (1) complex stimuli were taken from the album “Selected Songs” containing 35 lieder by Franz Schubert (Max Friedländer/C. F. Peters, Frankfurt/London/New York) accessed from the kern.hundrum.net website in the ^**^kern format (Huron, 1997); (2) simple stimuli were taken from the Church of England hymnal “Ancient and Modern” containing 120 hymns (Nicholson et al., 1950), previously used in Pearce et al. (2010a). For this purpose, the hymns had been rhythmically simplified by a skilled musicologist (see Pearce et al., 2010a for details). A number of pitch encoding errors found in the complex Schubert files were corrected with reference to the original scores. The complex corpus, furthermore, spanned a larger pitch range (A3-A5 in scientific pitch notation) than the simple corpus (C4-F5) and used a wider range of notes from the scale. We include the simple stimuli for comparison with previous research (Pearce et al., 2010a; Omigie et al., 2012) and the complex stimuli to test whether the findings generalize to more complex musical styles.

The Shannon entropy of the probability distributions estimated by the computational model described in Section A Computational Model of Auditory Expectation (see Pearce, 2005 for further details) was used to select stimuli for four experimental categories resulting from the factors *complexity* (two levels: complex/simple) and *entropy* (two levels: high/low). For selection of simple stimuli, the model represented each note as a pair of values: the first is pitch interval (the difference in semitones between consecutive notes) and the second, scale degree (the interval in semitones from the tonal center). For selection of the complex stimuli, which contained rhythmic structure, the model representation included an additional value corresponding to the contour of the temporal inter-onset intervals preceding the note in question (see Section A Computational Model of Auditory Expectation for further details on representations used by the model). Prior to making its predictions, the long-term sub-model had been trained on a set of 566 German folksongs from a subset (Fink, 1893) of the Essen Folksong Collection (Schaffrath, 1992, 1993), 185 chorale melodies harmonized by J. S. Bach (Riemenschneider, 1941), and 152 Nova Scotian songs and ballads (Creighton, 1966). The predictions of the short-term sub-model and long-term sub-model were combined to produce a single probability distribution predicting the pitch of the next note given the preceding sequence of notes (see Section A Computational Model of Auditory Expectation for further details about the long-term and short-term sub-models).

Seventy-two candidate notes were selected from the highest and lowest entropy predictions. For selection of candidates, the entropy values of the full distributions containing probability estimates for all of the 37 chromatically distributed pitches appearing in the training corpus (i.e., B2-B5) were used (“37-tone entropy”). Subsequent segmentation resulted in 72 candidate contexts corresponding to the 72 candidate notes each of which was preceded by a local context. These contexts always included a minimum of one complete phrase containing at least eight notes in total and making use of a minimum of four distinct pitches. Furthermore, candidate contexts always began with a note that started a phrase in the original song or hymn.

Nine probe tones (distributed with intervals of a semitone) were then assigned to each context. These were centered on the median pitch of the given context, but displaced so that actual continuation pitches were always included. This ensured that unrealistically high entropy values did not result from exclusion of highly expected continuations. Note durations of probe tones corresponded to those in the original melodies (cf. Schmuckler, 1989).

The 24 final stimuli contexts were ultimately selected using new entropy predictions based on probability distributions of the nine probe tones normalized so that each probability distribution summed to unity (“9-tone entropy”). While the initial selection of candidate contexts used the notated key signature, here the key signature used to compute the scale degree parameter of the model was computed using Temperley's (1999) enhanced implementation of the Krumhansl-Schmuckler algorithm (Krumhansl, 1990, pp. 77–110) so as to estimate the sense of musical key induced in typical listeners when listening to these particular melodic segments.

**Figure 2:**
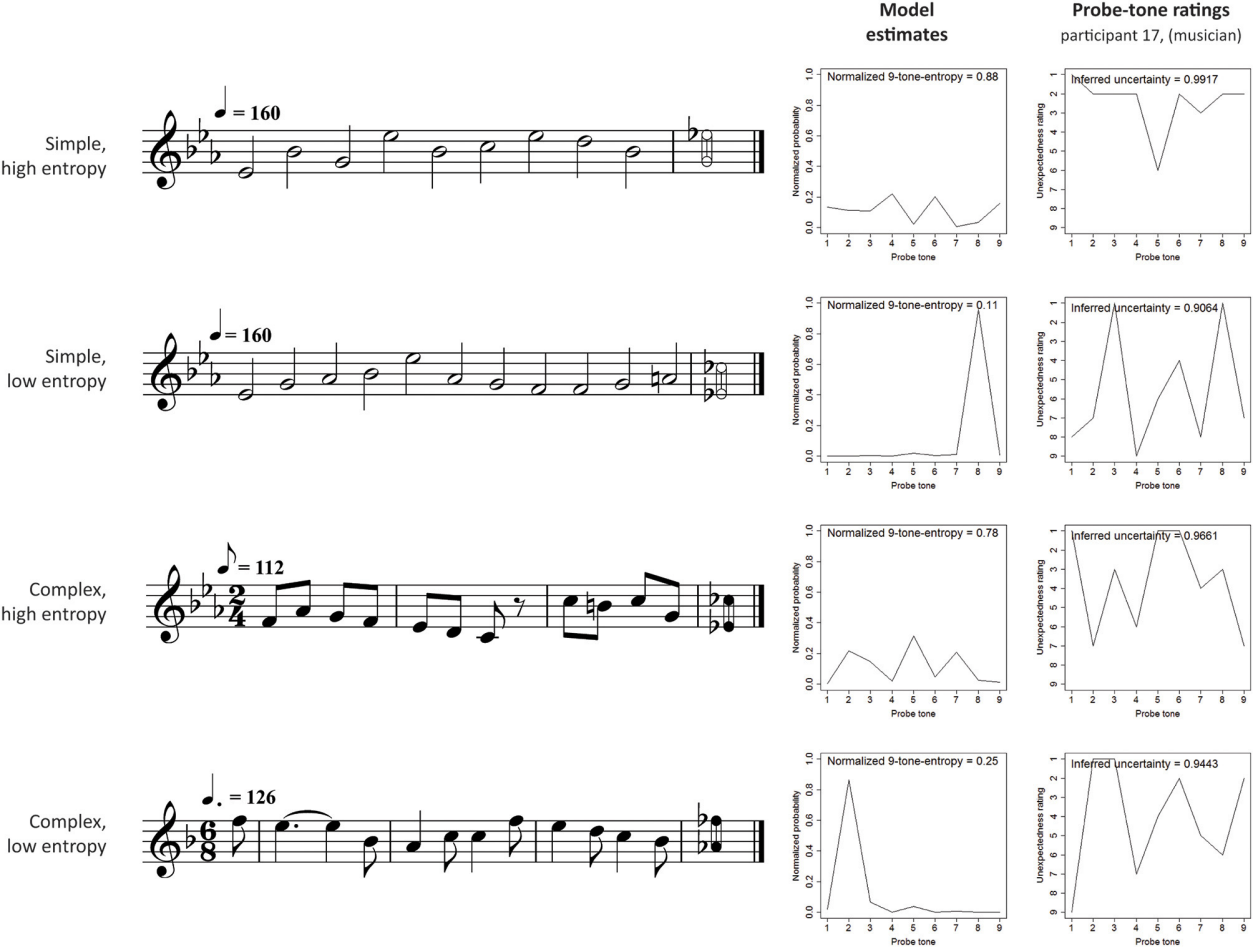
**Example stimuli in each experimental condition, with model output and one listener's probe-tone ratings**. There were 6 stimuli in each of the four conditions corresponding to a 2 × 2 factorial design. The stimuli are drawn from two musical styles, isochronous hymns (simple, *n* = 12) and Schubert lieder (complex, *n* = 12). For each style, 6 stimuli lead the model to make high-entropy predictions about the pitch of the next note and the other 6 produce low-entropy predictions. The high-entropy contexts produce relatively flat probability distributions over the set of 9 possible pitch continuations. The low-entropy contexts, on the other hand, produce “spiky” distributions in which one note has a higher probability of occurrence than the others. Using a 9-point scale, listeners rated how unexpected they found each of the 9 possible continuations in a probe-tone experiment. The figure shows the responses of participant 17 (from the musician group).

Stimuli contexts were exported from Sibelius 4 (Finn and Finn, 2005) as MIDI files using an acoustic piano sound. Simple stimuli were presented in a tempo within the normal range of this style (corresponding to crotchet = 160 beats per minute), and complex stimuli used the tempo of a standard recording (by Dietrich Fischer-Dieskau and Gerald Moore, Deutsche Grammophon, ADD 0289 477 8989) with the tempo increased by 20% to compensate for the lack of dynamic variation in the piano sound used here compared to the human voice. Figure 2 shows examples of four melodic contexts used in the experiment (one for each of the four conditions). The range of nine chromatically distributed probe tones and the probability estimates of the computational model are also shown for each melodic context.

### Procedure

Candidate participants were pre-screened for musical background and demographic data using an online survey containing the three subscales “musical training,” “importance,” and “emotion” from Gold-MSI (Müllensiefen et al., 2011). Eligible participants were subsequently tested individually using headphones. The complete paradigm lasted 60–90 min depending on individual pace and the extent of voluntary breaks taken. Participants provided informed written consent, and the experimental protocol had received prior approval from the Ethics Committee of the Department of Psychology, Goldsmiths, University of London.

In Phase 1, the 24 contexts were presented in randomized order without probe tones. Participants provided dichotomous (yes/no) familiarity judgments and rated explicit uncertainty on a 9-point Likert scale (1: “highly certain”; 9: “highly uncertain”). The exact question asked was “How certain [do] you feel about how the melody would continue?”; this was elaborated with a description stating that “if you are absolutely sure about how the melody would have continued, you respond 1” and “if you are completely unsure about how the melody would have continued and think it could equally well have continued in many different ways, then you respond 9.” Participants were instructed to use the full range of the scale. Data from familiar melodies were excluded from further analysis, thus ensuring that the results would reflect only schematic and not veridical influences on expectation and uncertainty.

In Phase 2, 216 sound files (i.e., 24 contexts each followed by nine probe tones) were presented in randomized order, and participants rated the unexpectedness of probe tones (1: “highly expected”; 9: “highly unexpected”; see Figure 2 for examples of probe-tone ratings from one participant). Inferred uncertainty data was obtained by taking the normalized entropy computed from the distributions of normalized unexpectedness ratings. Addressing possible closure effects, identified by Aarden (2003), participants were explicitly instructed “not [to] think of the last note as the ultimate note of the melody, but rather as a continuation tone after which more notes may or may not come.”

The two experimental phases were not counterbalanced because we did not want the actual continuations heard during Phase 2 to influence participants' judgments of explicit uncertainty in Phase 1. Before each experimental phase, a trial melody was played with the experimenter present, and an opportunity was provided to ask questions and adjust the sound level.

## Results

### Explicit uncertainty

**Figure 3:**
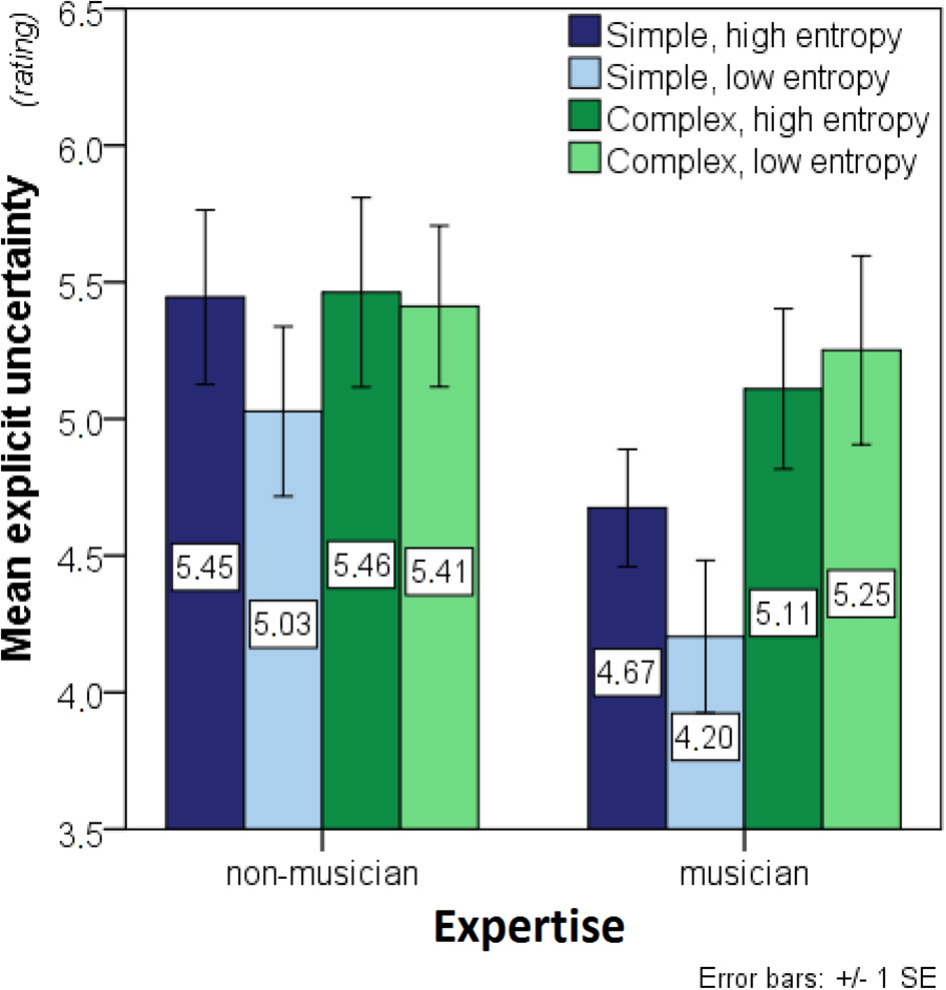
**Bar charts showing mean explicit uncertainty (before outlier exclusion)**. Significant main effects of complexity as well as interaction effects of expertise-by-complexity were found. The stimuli are distinguished in terms of: entropy (2 levels: high, low) returned when the model uses the stimulus as a context for predicting the next note; and complexity (2 levels: simple, complex) which refers to their musical style. Simple stimuli are isochronous hymn melodies while the complex stimuli are taken from Schubert lieder. Expertise (2 levels: musician, non-musician) refers to the level of musical training of the participants; musicians scored ≥ 33 on the musical training subscale of the Goldsmiths Musical Sophistication Index (Gold-MSI, v0.9) (Müllensiefen et al., 2011), whereas non-musicians scored ≤ 20 (see Section Participants).

A 2 × 2 × 2 ANOVA with *complexity* and *entropy* as within- and expertise as between-participant factors was run on the explicit uncertainty data. Prior to this, four outliers (two musicians and two non-musicians) were excluded to obtain normally-distributed data in all experimental conditions. Outliers were defined as values exceeding 1.5 times the interquartile range from the 1st or the 3rd quartile, and normality was confirmed by a Shapiro-Wilk test, all *W*_(15)_ ≥ 0.892, all *p* ≥ 0.07. Complex contexts, *F*_(1, 28)_ = 5.518, *p* < 0.03, produced higher uncertainty levels, and non-musicians were more uncertain than musicians, *F*_(1, 28)_ = 4.530, *p* = 0.04 (Figure 3). Although it was in the expected direction, the main effect of entropy did not reach significance *F*_(1, 28)_ = 1.492, *p* = 0.23.

A significant expertise-by-complexity interaction, *F*_(1, 28)_ = 4.640, *p* = 0.04, suggested that non-musicians were less sensitive to stylistic differences; moreover, a marginally non-significant complexity-by-entropy interaction, *F*_(1, 28)_ = 3.763, *p* = 0.06, suggested that the complex stimuli might not have produced the expected main effects of entropy.

The interaction effects justified two 2 × 2 *post-hoc* ANOVAs on the data from simple and complex stimuli separately. Whereas simple contexts showed significant effects of expertise, *F*_(1, 28)_ = 17.404, *p* < 0.01, and entropy, *F*_(1, 28)_ = 4.673, *p* = 0.04, complex contexts showed no effects of entropy, *F*_(1, 28)_ = 0.246, *p* = 0.62, or expertise, *F*_(1, 28)_ = 0.296, *p* = 0.59. These analyses suggest that the predicted effects of expertise and entropy on explicit uncertainty were only present for the simple stimuli.

Subsequently, the extent to which explicit uncertainty corresponded to the modeled entropy for each context was examined. For this analysis, averaging explicit uncertainty judgments across participants was warranted by high inter-individual consistency, Cronbach's α = 0.840. Since there is no reason to assume an upper bound on the number of pitch continuations represented by listeners, entropy values were computed from the full distributions comprising the probabilities of all 37 tones occurring across the musical corpus (i.e., “37-tone entropy”) using a single pitch feature (pitch interval linked with scale degree). Contrary to our predictions, non-parametric analysis (Spearman's *rho*) showed no significant correlation between average explicit uncertainty and entropy computed from modeled probabilities, *r*_*s*(22)_ = 0.199, *p* = 0.35.

Pearson's *r* from the correlation between each participant's explicit uncertainty and the modeled entropy across the 24 melodic contexts was then taken as a measure of the extent to which explicit uncertainty data from each participant corresponded to the model predictions (“explicit entropy-model-fit”). This measure spanned from *r*_(22)_ = −0.366 to *r*_(22)_ = 0.502 (*M* = 0.093; *SD* = 0.214) and a one-sample *t*-test showed that it was significantly different from zero, *t*_(33)_ = 2.537, *p* = 0.02. It did not differ between musicians (*M* = 0.071; *SD* = 0.263) and non-musicians (*M* = 0.115; *SD* = 0.157), *t*_(26.2)_ = 0.597, *p* = 0.56. When explicit entropy-model-fit (Fisher *Z*-transformed) was computed across contexts for each participant separately, no association was found with any of the Gold-MSI subscales, all *p* ≥ 0.28.

### Inferred uncertainty

For analysis of inferred uncertainty, we conducted a 2 × 2 × 2 ANOVA with *complexity* and *entropy* as within- and *expertise* as between-participant factors after exclusion of five outliers to obtain normally-distributed data in all experimental conditions, all *W* ≥ 0.862, all *p* ≥ 0.05. Because the removed outliers comprised five non-musicians and no musicians, thus causing a difference in group size, Levene's test was used to check for inequality of error variances after outlier exclusion. This test showed inequal error variances for the complex, low-entropy condition, *F*_(1, 27)_ = 6.313, *p* = 0.02, but not for the other three conditions, all *F*'s_(1, 27)_ ≤ 3.880, all *p*'s ≥ 0.06. Keeping this in mind, we proceeded with parametric analysis excluding the aforementioned outliers, but also decided to check our findings using non-parametric procedures.

**Figure 4:**
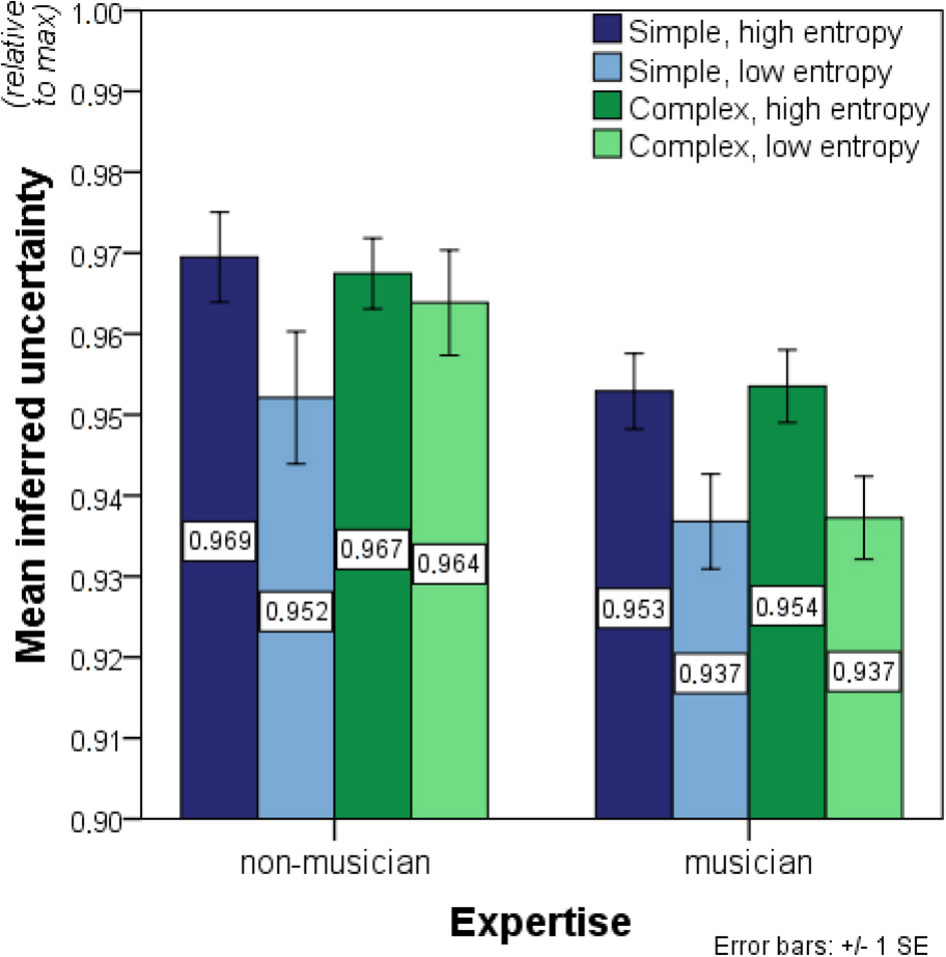
**Bar charts showing mean inferred uncertainty (i.e., the normalized entropy value of expectedness distributions before transformation and outlier exclusion)**. Significant main effects of entropy and expertise on inferred uncertainty were found as well as significant entropy-by-expertise and complexity-by-entropy interactions. No other main or interaction effects were present in the data. See legend to Figure 3 for further details.

The ANOVA on inferred uncertainty data revealed strongly significant effects of entropy on inferred uncertainty in the expected direction, *F*_(1, 27)_ = 37.529, *p* < 0.01 (Figure 4). Additionally, musicians showed significantly less inferred uncertainty than non-musicians, *F*_(1, 27)_ = 13.491, *p* < 0.01. No significant effects of complexity, *F*_(1, 27)_ = 0.440, *p* = 0.51, were found. Significant interaction effects were present for entropy-by-expertise, *F*_(1, 27)_ = 5.543, *p* = 0.03, and complexity-by-entropy, *F*_(1, 27)_ = 4.383, *p* < 0.05, but not for expertise-by-complexity, *F*_(1, 27)_ = 0.225, *p* = 0.64. Moreover, the three-way interaction complexity-by-entropy-by-expertise was significant, *F*_(1, 27)_ = 4.554, *p* = 0.04.

These interaction effects justified two *post-hoc* 2 × 2 ANOVAs on the simple and complex contexts separately with entropy as a within-participant factor and expertise as a between-participant factor. For the simple contexts, significant main effects of entropy, *F*_(1, 27)_ = 23.869, *p* < 0.01, and expertise, *F*_(1, 27)_ = 8.446, *p* < 0.01, were found. The entropy-by-expertise interaction, on the other hand, remained non-significant, *F*_(1, 27)_ = 0.010, *p* = 0.92. The same significant main effects of entropy, *F*_(1, 27)_ = 7.779, *p* < 0.01, and expertise, *F*_(1, 27)_ = 14.220, *p* < 0.01, were found for the complex contexts. Here, however, an additional significant entropy-by-expertise interaction was present, *F*_(1, 27)_ = 13.979, *p* < 0.01. Thus, the entropy-by-expertise interaction discovered in the initial omnibus test was primarily due to different response patterns for the complex contexts where only musicians showed systematic effects of entropy. Additionally, domain-relevant expertise had the strongest impact for low-entropy contexts.

Acknowledging the restriction in sample size caused by exclusion of five non-musicians in the initial analysis as well as the resulting inequality of error variances in the high-complexity conditions, a subsequent 2 × 2 × 2 ANOVA was run on a rank-transformed version of the full dataset, following the procedure suggested by Conover and Iman (1981). This analysis confirmed the previous results showing significant effects of entropy, *F*_(1, 32)_ = 52.383, *p* < 0.01, expertise, *F*_(1, 32)_ = 9.002, *p* < 0.01, entropy-by-expertise, *F*_(1, 32)_ = 4.777, *p* = 0.04, complexity-by-entropy, *F*_(1, 32)_ = 4.731, *p* = 0.04, as well as a significant three-way interaction, *F*_(1, 32)_ = 5.831 *p* = 0.02. Effects of complexity, *F*_(1, 32)_ = 0.528, *p* = 0.47, and expertise-by-complexity, *F*_(1, 32)_ = 0.716, *p* = 0.40, remained non-significant. It should be noted, however, that the application of Conover and Iman's rank transformation procedure to 2 × 2 × 2 multifactorial designs is not without its problems (Sawilowsky et al., 1989).

**Figure 5:**
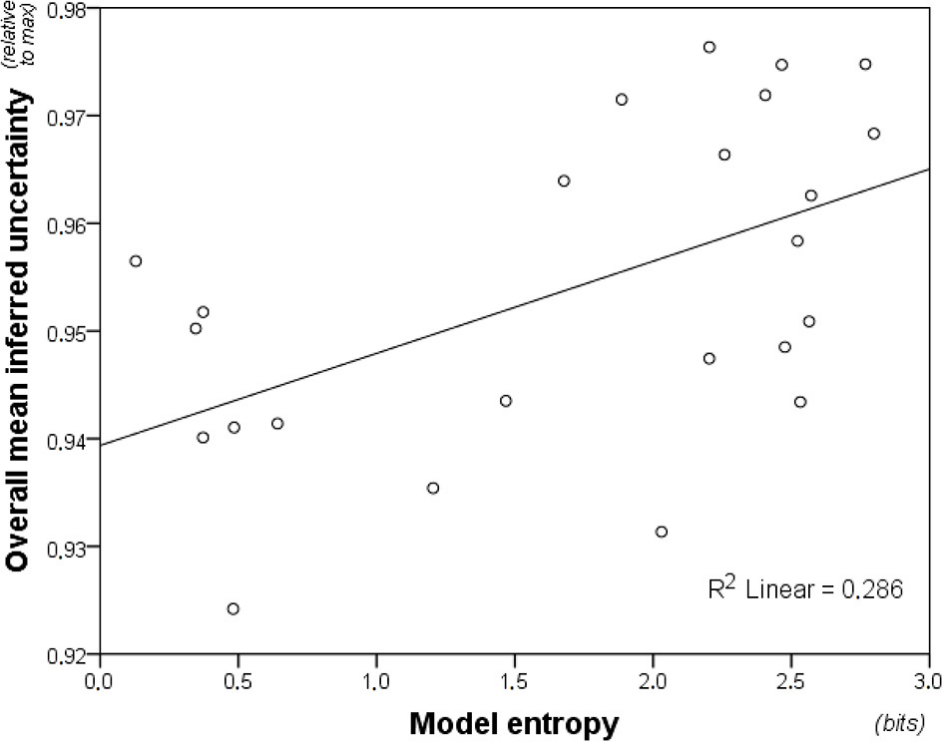
**Model entropy for the 24 melodic contexts plotted against inferred uncertainty averaged across participants**. The figure shows the relationship between the model predictions (entropy) and the uncertainty of the listeners. Inferred uncertainty correlates weakly, but significantly, with entropy predictions of the model. The model is a variable-order model combining both short-term and long-term sub-models.

High inter-individual consistency, Cronbach's α = 0.941, warranted averaging inferred uncertainty across participants. Average inferred uncertainty correlated overall with entropy (Figure 5), *r*_*s*(22)_ = 0.466, *p* = 0.02. Surprisingly, this was primarily driven by non-musicians, *r*_*s*(22)_ = 0.466, *p* = 0.02, with the correlation remaining marginally non-significant for musicians, *r*_*s*(22)_ = 0.345, *p* = 0.10. However, William's *t*-test, comparing dependent correlations of variables regressed on a common variable (Steiger, 1980), showed no significant difference between parametric correlation coefficients for the two groups, *t*_(21)_ = 0.885, *p* = 0.39.

As for explicit uncertainty above, inferred entropy-model-fit was computed by taking Pearson's *r* from the correlation between each participant's inferred uncertainty and the modeled entropy across the 24 melodic contexts. This measure spanned from *r*_(22)_ = −0.087 to *r*_(22)_ = 0.596 (*M* = 0.264; *SD* = 0.197) and a one-sample *t*-test showed that it was significantly different from zero, *t*_(33)_ = 7.791, *p* < 0.01. However, although musicians obtained higher inferred entropy-model-fit (*M* = 0.279; *SD* = 0.183) than non-musicians (*M* = 0.248; *SD* = 0.215) on average, this difference remained non-significant, *t*_(32)_ = −0.453, *p* = 0.65. Inferred entropy-model-fit (Fisher *Z*-transformed) did not correlate significantly with the Gold-MSI subscales for “musical training,” *r*_*s*(32)_ = 0.222, *p* = 0.21, “importance,” *r*_*s*(32)_ = 0.044, *p* = 0.81, or “emotion,” *r*_*s*(32)_ = −0.048, *p* = 0.79.

### Relationship between explicit and inferred measures of uncertainty

To establish whether inferred uncertainty represents a meaningful way of assessing predictive uncertainty in melodic expectation, we examine the relationship between explicit and inferred uncertainty. Parametric correlation analysis confirmed that average explicit uncertainty correlated significantly with average inferred uncertainty across all participants, *r*_(22)_ = 0.629, *p* < 0.01. Moreover, when analyzed separately, this was both the case for musicians, *r*_(22)_ = 0.513, *p* = 0.01, and non-musicians, *r*_(22)_ = 0.448, *p* = 0.03. Thus, explicit and inferred uncertainty are indeed related, albeit slightly less so for non-musicians than for musicians.

### Unexpectedness ratings

Having examined the relationship between model entropy and predictive uncertainty (both explicit and inferred), we turn now to an analysis of the unexpectedness ratings underlying the measure of inferred uncertainty. In particular, following previous research (e.g., Pearce et al., 2010a), we examine the relationship between model information content and the unexpectedness of single pitch continuations to the melodic contexts. First, however, we examine whether our experimental manipulations of entropy, musical training and structural complexity had an impact on the unexpectedness ratings.

**Figure 6:**
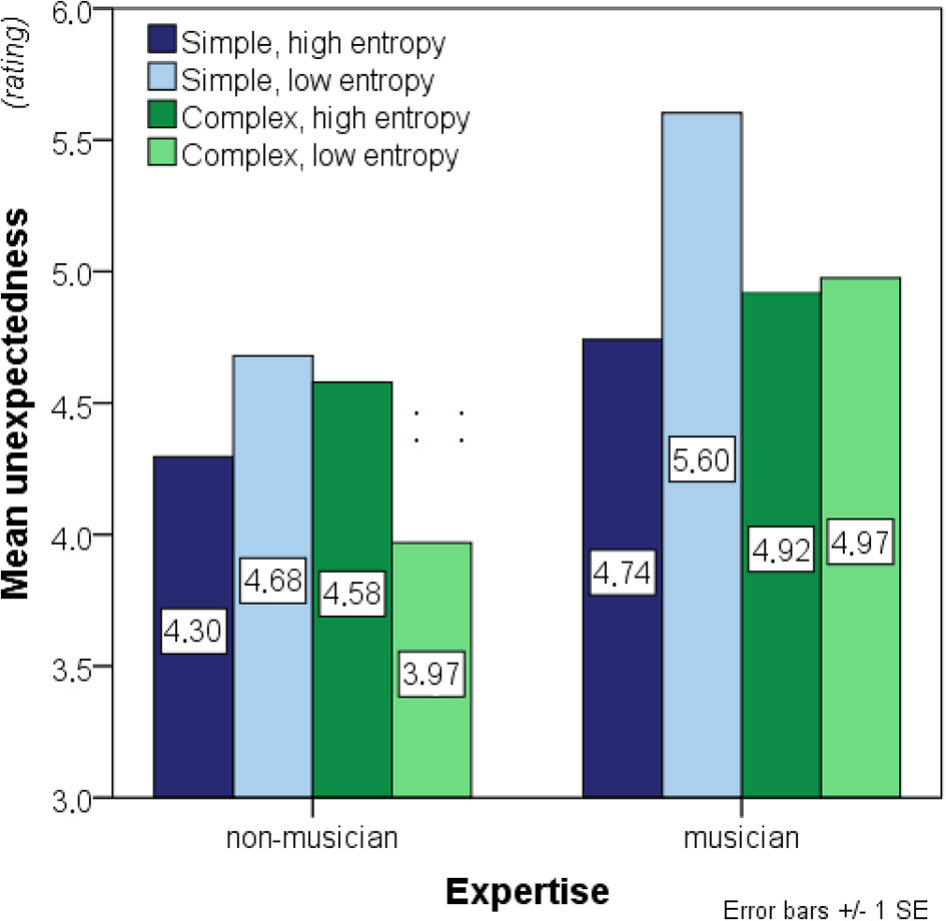
**Bar charts showing mean unexpectedness (before outlier exclusion)**. Significant main effects of entropy and complexity as well as interaction effects of entropy-by-expertise and complexity-by-entropy were found. See legend to Figure 3 for further details.

A 2 × 2 × 2 ANOVA was run on the unexpectedness ratings, using *entropy* and *complexity* as within-participant factors and *expertise* as a between-participant factor (see Figure 6). Prior to analysis, three outliers were excluded comprising one non-musician and two musicians to obtain normality in all experimental conditions, as confirmed by Shapiro-Wilk's test, all *W* ≥ 0.898, all *p* ≥ 0.09. Levene's test was used to check for inequality of error variances after outlier exclusion due to the resulting difference in group size. Equal error variances were found for three of the four conditions, all *F*'s_(1, 29)_ ≤ 1.263, all *p*'s ≥ 0.27, but not for the conditions with complex low-entropy contexts, *F*_(1, 29)_ = 5.272, *p* < 0.03.

A significant entropy-by-expertise interaction, *F*_(1, 29)_ = 17.677, *p* < 0.01, was present. Additionally, a complexity-by-entropy interaction, *F*_(1, 29)_ = 53.287, *p* < 0.01 was found, but no other interactions reached significance, both *F*'s ≤ 1.346, *p* ≥ 0.26. Main effects were found of entropy, *F*_(1, 29)_ = 5.508, *p* = 0.03, expertise, *F*_(1, 29)_ = 6.848, *p* = 0.01, and complexity, *F*_(1, 29)_ = 4.561, *p* = 0.04 (Figure 6). This suggests that musicians experienced the melodic continuations as more unexpected than non-musicians and that low-entropy contexts evoked greater unexpectedness than did high-entropy contexts. Furthermore, musicians seemed to respond differently to entropy differences than non-musicians by rating continuation tones to low-entropy contexts as more unexpected on average. It also appears that the difference between low- and high-entropy contexts in overall unexpectedness was primarily driven by the structurally simple stimuli.

The interaction effects were further investigated with two separate 2 × 2 ANOVAs on simple- and complex contexts separately with expertise as a between-participants factor and entropy as a within-participants factor. This analysis confirmed that musicians generally experienced melodic continuations as more unexpected than non-musicians for both the simple, *F*_(1, 29)_ = 5.087, *p* = 0.03, and the complex contexts, *F*_(1, 29)_ = 6.303, *p* = 0.02. Main effects of entropy were still present for simple, *F*_(1, 29)_ = 101.297, *p* < 0.01, and for complex contexts, *F*_(1, 29)_ = 7.067, *p* = 0.01, and the entropy-by-expertise effect also remained significant for simple, *F*_(1, 29)_ = 11.407, *p* < 0.01, and complex contexts, *F*_(1, 29)_ = 9.544, *p* < 0.01. Importantly, however, the directions of the entropy and the entropy-by-expertise effects were not consistent between the two complexity conditions. For the simple contexts, as hypothesized, low-entropy contexts were perceived as more unexpected on average, and this effect was stronger in musicians than in non-musicians. For the complex contexts, on the other hand, musicians did not on average respond differently to low- than to high-entropy contexts whereas non-musicians actually perceived low-entropy contexts as more expected on average than high-entropy contexts.

**Figure 7:**
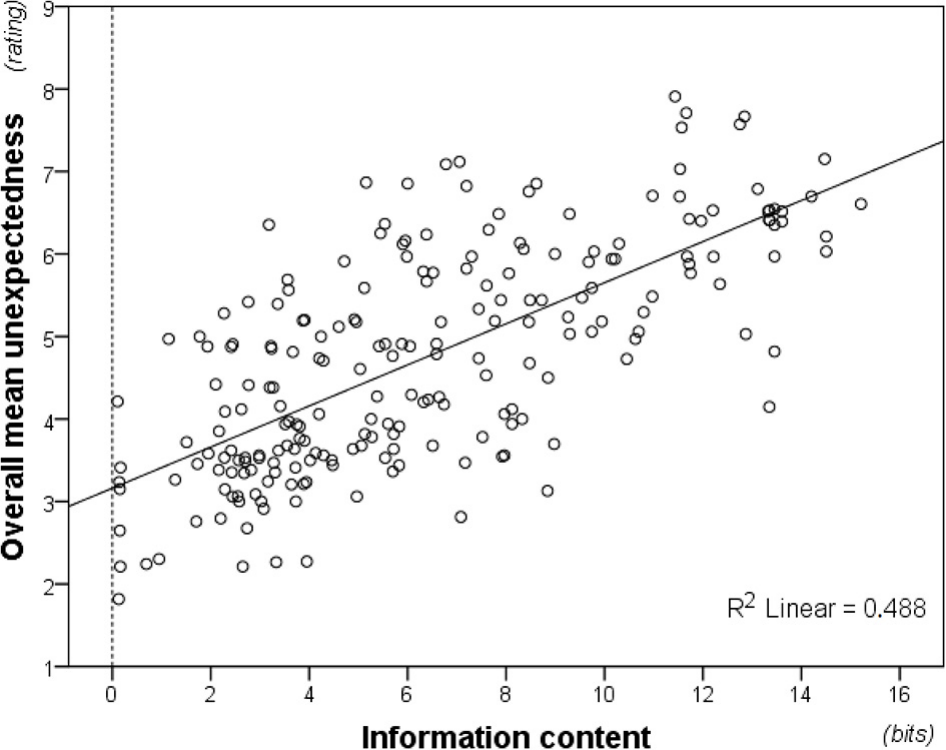
**Information content (modeled from the full pitch alphabet; i.e., 37-tone information content) plotted against unexpectedness ratings averaged across participants**. Perceived unexpectedness increases with information content.

Averaging unexpectedness ratings across participants was warranted by high inter-individual consistency, Cronbach's α = 0.973. As predicted, the averaged ratings correlated strongly with modeled information content (Figure 7), *r*_*s*(214)_ = 0.695, *p* < 0.01. Moreover, this correlation was significant both for musicians, *r*_*s*(214)_ = 0.701, *p* < 0.01, and non-musicians, *r*_*s*(214)_ = 0.569, *p* < 0.01, and William's *t*-test established that musicians produced a significantly better fit to the model, *t*_(213)_ = 3.455, *p* < 0.01.

**Figure 8:**
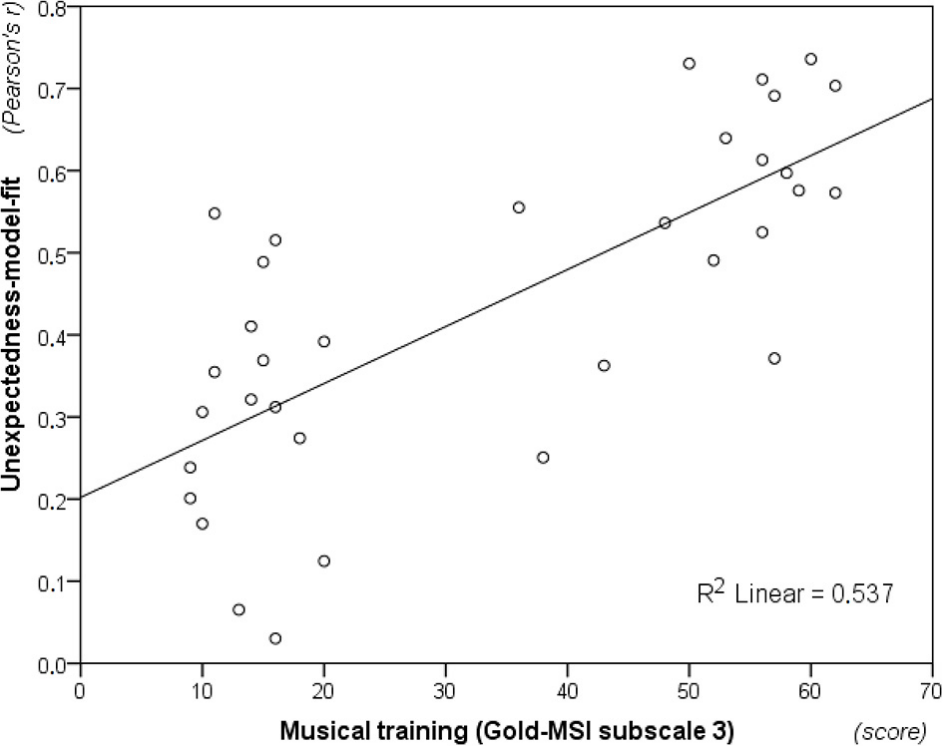
**Musical training (i.e., subscale 3 from *Goldsmiths Musical Sophistication Index*, v. 0.9) plotted against unexpectedness-model-fit (i.e., Fisher *Z*-transformed correlation coefficient between model information content and unexpectedness ratings, see Section Unexpectedness Ratings for details)**. The fit of unexpectedness data to the entropy predictions of the model increases with levels of musical training.

Finally, unexpectedness-model-fit was computed by taking Pearson's *r* from the correlation between each participant's unexpectedness ratings and the modeled information content across the 216 melodic contexts from Phase 2 of the experiment. This measure spanned from *r*_(214)_ = 0.030 to *r*_(214)_ = 0.627 (*M* = 0.397; *SD* = 0.165), and a one-sample *t*-test showed that it was significantly different from zero, *t*_(33)_ = 14.048, *p* < 0.01. Moreover, musicians (*M* = 0.508; *SD* = 0.107) scored significantly higher than non-musicians (*M* = 0.287; *SD* = 0.137), *t*_(32)_ = 5.230, *p* < 0.01. Despite significant bivariate correlations between unexpectedness-model-fit (Fisher *Z*-transformed) and the Gold-MSI subscales for musical training” (Figure 8), *r*_*s*(32)_ = 0.728, *p* < 0.01, importance of music, *r*_(32)_ = 0.548, *p* < 0.01, and emotional engagement with it, *r*_(32)_ = 0.364, *p* = 0.03, subsequent multiple regression analysis (using a backward stepwise procedure with a removal criterion corresponding to the probability of *F* ≥ 0.10) revealed that only musical training contributed in explaining a significant proportion of the variance, *R*^2^ = 0.537, *R*^2^_adj._ = 0.522, *F*_(1, 32)_ = 37.057, *p* < 0.01.

### Model comparisons

Finally, model comparisons were made to assess which model parameters (Pearce, 2005; see Section A Computational Model of Auditory Expectation) maximize fit between entropy and listeners' uncertainty (explicit and inferred), and also between information content and listeners' expectedness. Probability distributions were generated for all stimuli using models that varied in terms of the order-bound on the Markov or *n*-gram model (order: *n*−1 ≤ {0, 1, 2, 3, 4}, variable-order) and the system configuration (short-term sub-model only, long-term sub-model only, or both sub-models combined). Non-parametric correlation coefficients were computed across stimuli between the model estimates and the empirical data (unexpectedness, inferred uncertainty, explicit uncertainty) averaged across all participants and also separately for musicians and non-musicians.

We also compare these results to other competing models in the literature. For the uncertainty data, following Schmuckler (1989), difference scores were computed between the average and the minimum information content for any given distribution obtained with the standard model configuration (both sub-models combined, variable order). For the unexpectedness data, we tested an implementation (Schellenberg, 1997) of Narmour's (1990, 1992) Implication-Realization Model with three predictors entered into a multiple regression analysis: proximity (Schellenberg, 1996), pitch reversal (Schellenberg, 1997), and tonal hierarchy (Krumhansl, 1995b).

As shown in Table 1, higher correlation coefficients were obtained using entropy and information content values from the probabilistic model than for the corresponding competing model in all cases but one (inferred uncertainty for the non-musicians). Regarding the probabilistic model itself, implementations with a maximum order bound of 1 were superior in predicting unexpectedness ratings whereas higher maximum order bounds of 3 and variable order produced the highest correlations with inferred and explicit uncertainty, respectively. The short-term sub-model, which uses only the local context, showed non-significant correlations with the listeners' responses, which is perhaps unsurprising for the short melodic excerpts used. The configuration combining both sub-models showed no advantage over the long-term sub-model, which is trained through exposure to a large corpus of melodies.

**Table 1:**
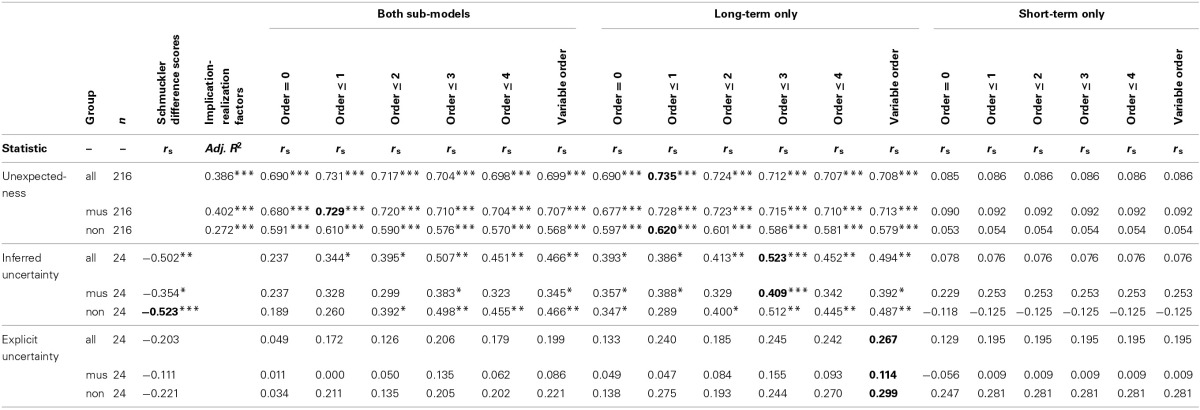
**Comparison of model-fit for different parameter values of the computational model of auditory expectation (Pearce, 2005) as well as for a three-factor implementation of the Implication-Realization Model (Schellenberg, 1997) and for Schmuckler difference scores (Schmuckler, 1989)**.

Model-fit corresponds to the correlation between modeled information content or entropy computed from the full 37-tone distributions with the unexpectedness or uncertainty data from the listeners, respectively. Highest model-fit for each empirical dataset is indicated in bold. The implementations of the model comprised configurations with different bounds on the maximum order of n-grams making predictions based only on the present context (short-term sub-model only), only on the training corpus (long-term sub-model only), or on a combination thereof (both sub-models combined). The Implication-Realization factors included proximity (Schellenberg, 1996), pitch reversal (Schellenberg, 1997), and tonal hierarchy (Krumhansl, 1995b), and difference scores were based on the difference between the minimum and average 37-tone information content for a given probability distribution estimated by the model configured with both sub-models combined using variable order. ^*^p < 0.1; ^**^p < 0.05; ^***^p < 0.01.

## Discussion

Our goal was to assess Shannon entropy as a model of predictive uncertainty in music cognition. Structurally *simple* and *complex* stimuli were created by taking melodies from English hymns and lieder by Franz Schubert, respectively. Entropy was estimated by a computational model of expectation (Pearce, 2005; see Section A Computational Model of Auditory Expectation for details) and used to select high and low-entropy contexts. Listeners' predictive uncertainty was elicited in two ways: first, by explicit judgments (explicit uncertainty); and second, by computing the Shannon entropy of subjective expectedness distributions obtained using the probe-tone method (inferred uncertainty). Below, we consider the implications of the findings with respect to each of our four initial hypotheses.

Regarding the first hypothesis, melodic contexts with high entropy were experienced as more uncertain than low-entropy contexts, for inferred uncertainty. Furthermore, model entropy correlated significantly with inferred uncertainty averaged across participants. Model comparisons suggested that this effect was driven largely by the long-term sub-model reflecting schematic expectations rather than short-term learning from the individual stimuli, using context lengths of up to 3. Comparisons between entropy and Schmuckler's (1989) method of estimating uncertainty applied to the model distributions showed that entropy provides a better fit to the data in most cases. Although there was a significant correlation between explicit and inferred uncertainty, an overall effect of entropy on explicit uncertainty was only apparent for the simple stimuli. Furthermore, explicit uncertainty data averaged across participants did not correlate significantly with model entropy (or with uncertainty computed from the model output using the Schmuckler method). These results suggest that uncertainty can be characterized in terms of the properties of conditional probability distributions, learned through exposure to music. However, this probabilistic knowledge does not become fully available to conscious introspection.

As predicted by our second hypothesis, musicians showed lower inferred uncertainty than non-musicians for both levels of complexity and also lower explicit uncertainty specifically for the structurally simple stimuli. For the complex melodic stimuli the effect of expertise on inferred uncertainty was greater in low-entropy contexts. These results suggest that musicians are able to generate expectations for particular melodic continuations more effectively than non-musicians. This may be a result of formal musical training or simply having listened to more music, in both focused and incidental situations, providing greater exposure for implicit learning (Pearce, 2014). Another possibility is that musical training enhances attention to music, which in turn improves pitch processing (Jones et al., 2006) and the efficiency of statistical learning (Toro et al., 2005). Research is required to tease apart these interpretations.

The data are also consistent with our third hypothesis, predicting an entropy-by-expertise interaction for the unexpectedness ratings. The probe tones continuing low-entropy contexts have lower probability on average than the probe tones continuing high-entropy contexts and, therefore, low-entropy contexts would be expected to show greater unexpectedness on average than high-entropy contexts. The results show that this effect was larger for musicians than for non-musicians. This is important because previous probe-tone studies have not systematically manipulated entropy. High-entropy contexts do not afford the possibility of strong specific expectations, therefore both musicians and non-musicians tend to rate all continuations as being equally likely. In these contexts, there is no advantage to be gained over a default model predicting each possible continuation with equal probability. In other words, the cognitive model of the non-musician (which tends toward such a default model), generating expectations with relatively high entropy, performs equally well in this context as the more finely tuned model of the expert musician. In low-entropy contexts, however, musicians are able to generate sharper and more specific expectations for the different possible continuations than non-musicians, reflected in the fact that they show greater unexpectedness for low-probability continuations than non-musicians. Again this effect was stronger for the structurally simple stimuli, suggesting that other factors may impact on the accuracy of the musician's optimized predictive model.

Turning to our fourth hypothesis, the results consolidate earlier findings that expectedness decreases with increasing information content (Pearce and Wiggins, 2006; Pearce et al., 2010a; Omigie et al., 2012). In our present results, this effect was shown to generalize across the two levels of rhythmic and tonal complexity and contexts that were systematically different in terms of entropy. The fact that non-musicians show this effect is consistent with the hypothesis that statistical learning occurs automatically in line with previous research showing that adults and infants internalize transitional probabilities in syllable sequences even when these are presented incidentally while creating computer illustrations (Saffran and Newport, 1997). More importantly, however, the present study demonstrated for the first time that the fit between perceived unexpectedness and information content is greater for musicians than non-musicians, and interestingly, increases linearly with degree of musical training. Model comparisons showed that listeners' expectedness ratings correlated most highly with a long-term model using contexts of one note, suggesting that expectations are driven by low-order, schematic predictions derived from long-term exposure to music rather than short-term learning from the individual stimuli. Importantly, expectedness ratings correlated more highly with the probabilistic model than with a competing rule-based model (Narmour, 1990; Schellenberg, 1997).

We have conducted detailed model comparisons including both different parameterizations of the probabilistic model of auditory expectation (Pearce, 2005) and other models in the literature (Schmuckler, 1989; Schellenberg, 1997). The results indicate that the probabilistic model accounts for the listener's expectedness ratings better than Schellenberg's (1997) implementation of the Implication-Realization Model (Narmour, 1990) for musicians and non-musicians. Overall, the long-term sub-model fit the data much better than the short-term sub-model demonstrating that listeners' expectations reflect the overall statistical structure of Western tonal music rather than the statistical structure of each melodic stimulus. The optimal order-bound was 1, suggesting that listeners' expectations are based on a context of one note only, although higher-order models also produce strong correlations with only slightly lower correlation coefficients. For the inferred uncertainty data, the best performing model was again the long-term sub-model, but this time with an order bound of 3, suggesting that listeners generate expectancy distributions using a context of three notes. We compared entropy with a method of computing uncertainty used by Schmuckler (1989), consisting of the difference between the average and the minimum probability in the distribution returned by the model (standard configuration: both sub-models combined, variable order). This method produced a slightly higher correlation than the order-3 long-term sub-model for the non-musicians but not for the musicians or for the data set as a whole. None of the models produced a significant correlation with the explicit uncertainty data.

Several directions remain open for future research. The relatively small sample size (24 contexts) may have resulted in Type II errors, therefore replication with larger samples is important. Furthermore, the results should be replicated using other methodological approaches to assess uncertainty including reaction time studies (e.g., Bharucha and Stoeckig, 1986; Bigand et al., 2001, 2005; Tillmann and Bharucha, 2002; Bigand and Poulin-Charronnat, 2006; Tillmann et al., 2006; Omigie et al., 2012) and various methods for assessing expectations continuously throughout a listening session without pausing the stimulus to collect responses (e.g., Eerola et al., 2002; Aarden, 2003; Toiviainen and Krumhansl, 2003; Pearce et al., 2010a). There is a difficulty with the latter in that changing the pitch of one note effectively changes the size of two pitch intervals, making it difficult to ascertain whether participants' responses relate to the note itself or the interval following it.

The weaker findings for explicit uncertainty might indicate that listeners had difficulty understanding the instructions. They were given practice trials and an opportunity to ask questions, and none indicated any difficulty. It is possible that focusing their attention more specifically on pitch category of the next note in the melody would produce more sharply defined responses. We think it is likely that listeners had difficulty introspecting about their prospective sense of uncertainty, suggesting that implicit behavioral measures or physiological research might be fruitful avenues for future investigation.

The model comparisons suggest that listeners' predictive uncertainty and expectedness for particular tones most closely resembled the output of the long-term sub-model with contexts ranging from 1 to 3 notes. This suggests a greater influence of relatively low-order conditional probabilities derived from long-term schematic exposure rather than short-term learning from the individual stimuli. However, both of these effects may result at least in part from the relatively short contexts used as stimuli. Furthermore, it is possible that the features and datasets chosen to train the model do not fully capture listeners' cognitive representations. Therefore, further research is needed on the question of how much context and which representations listeners use when generating melodic expectations. Although the results generalized across the two musical styles, they were stronger and more consistent for the simple musical style. Further work is required to tease apart and model the different musical components (pitch, tonality, rhythm, interactions of these features) driving the effects of musical complexity on the perception of uncertainty.

We interpret our findings in terms of a Statistical Learning Hypothesis, suggesting that schematic expectations reflect probabilistic relationships between sensory events learned implicitly through exposure. The results are also consistent with predictive coding theory (Friston, 2005, 2009, 2010), which postulates that bottom-up sensory perception is guided by hierarchical top-down predictive mechanisms. Predictions arise from cognitive/neural representations of the environment and serve to interpret and disambiguate the incoming sensory data. These predictions are continuously optimized through a recursive process of learning through monitoring of prediction errors, corresponding to discrepancies recorded between top-down-generated predictions and incoming sensory input, which guide ongoing neuronal micro-plasticity which minimizes further prediction errors.

Research in cognitive neuroscience suggests that prediction errors to unexpected auditory events are stronger in musicians than in non-musicians. The Mismatch Negativity (MMN) represents an ERP component appearing in response to a deviant stimulus occurring in a sequence of regular stimuli. There is evidence that the MMN has higher amplitude in musicians than in non-musicians (Näätänen et al., 2007) and that it varies as a function of the musical style that musicians have specialized in Vuust et al. (2012). For musicians, larger MMNm amplitudes have been found in response to interval and contour violations of brief melodic phrases (Fujioka et al., 2004), and larger left-lateralized MMNm amplitudes also seem to result in response to deviant rhythms (Vuust et al., 2005, 2009). Furthermore, MMN responses to slightly mistuned major chords were present only in professional violinists and not in non-musicians (Koelsch et al., 1999), and MMN responses to omission of tones were found in musicians, but remained smaller or absent in non-musicians (Rüsseler et al., 2001). Research with EEG/MEG moreover shows that ERP/ERF components distinguishing high- and low-probability events in music are influenced by musical training (Loui et al., 2009; Pearce et al., 2010a; Kim et al., 2011). In an MEG study using chord sequences as stimuli, Kim et al. (2011) found interaction effects of musical training by conditional probability on the Early Anterior Negativity (EANm) amplitude.

Drawing on these theoretical frameworks and results, we hypothesize that predictive uncertainty depends on internal cognitive models of the sensory environment, which generate conditional probability distributions predicting the next event in a sequence given the preceding events and which are optimized through experience. We hypothesize that the musicians in our studies possess more accurate cognitive models, which are able to take advantage of the low-entropy contexts to generate distributions with strong, specific predictions. The non-musicians are less able to generate highly certain predictions in these contexts. For the high-entropy stimuli, which do not allow specific predictions based on schematic learning of musical structure, the musicians show no advantage over the non-musicians, because the stimulus does not allow them to take advantage of their optimized cognitive models. The results suggest that the accuracy of the predictive model is also affected by the structural complexity of the stimulus, with stronger and more consistent effects of entropy for simple stimuli, especially for the non-musicians. Furthermore, the results were stronger and more consistent for inferred uncertainty than for explicit judgments of predictive uncertainty, suggesting that listeners may not have full conscious access to underlying probabilistic knowledge influencing the predictive uncertainty of their expectations.

The results contribute to a larger body of research aiming to develop a general cognitive account of predictive sequence processing (Conway and Christiansen, 2005; Friston, 2005, 2009, 2010; Hale, 2006; Bar, 2007, 2011; Bubic et al., 2010). Future work should investigate whether the relationships established here between Shannon entropy and predictive uncertainty generalize beyond music to other complex sequential auditory domains such as language, to perception of visual sequences as well as to multimodal sequence perception. Furthermore, the results add to an ongoing discussion about the impact of explicit training on implicit learning (e.g., Mathews et al., 1989; Willingham and Goedert-Eschmann, 1999; Farrow and Abernethy, 2002; Sun et al., 2005). Further research is required to examine which aspects of training (increased attention, increased exposure, or explicit knowledge) are responsible for the effects we observe here and how domain-specific these effects are.

## Author contributions

Both authors contributed equally to the research reported here.

### Conflict of interest statement

Despite financial support from EPSRC, EPSRC had no role in the design of the study, the data collection, analysis, data interpretation, writing of the report or in the decision to submit the paper for publication. The authors declare that the research was conducted in the absence of any commercial or financial relationships that could be construed as a potential conflict of interest.

## Footnotes

^1^Documentation and downloads are available here: http://code.soundsoftware.ac.uk/projects/idyom-project
